# Epstein-Barr Virus and Neurological Diseases

**DOI:** 10.3389/fmolb.2021.816098

**Published:** 2022-01-10

**Authors:** Nan Zhang, Yuxin Zuo, Liping Jiang, Yu Peng, Xu Huang, Lielian Zuo

**Affiliations:** ^1^ Department of Physiology, Institute of Neuroscience Research, Hengyang Key Laboratory of Neurodegeneration and Cognitive Impairment, Hengyang Medical College, University of South China, Hengyang, China; ^2^ Hunan Dongkou People’s Hospital, Shaoyang, China

**Keywords:** epstein-barr virus, neuroinflammation, multiple sclerosis, alzheimer’s disease, immunoreaction, EBNA1, LMP1

## Abstract

Epstein-Barr virus (EBV), also known as human herpesvirus 4, is a double-stranded DNA virus that is ubiquitous in 90–95% of the population as a gamma herpesvirus. It exists in two main states, latent infection and lytic replication, each encoding viral proteins with different functions. Human B-lymphocytes and epithelial cells are EBV-susceptible host cells. EBV latently infects B cells and nasopharyngeal epithelial cells throughout life in most immunologically active individuals. EBV-infected cells, free viruses, their gene products, and abnormally elevated EBV titers are observed in the cerebrospinal fluid. Studies have shown that EBV can infect neurons directly or indirectly *via* infected B-lymphocytes, induce neuroinflammation and demyelination, promote the proliferation, degeneration, and necrosis of glial cells, promote proliferative disorders of B- and T-lymphocytes, and contribute to the occurrence and development of nervous system diseases, such as Alzheimer’s disease, Parkinson’s disease, multiple sclerosis, acute cerebellar ataxia, meningitis, acute disseminated encephalomyelitis, and brain tumors. However, the specific underlying molecular mechanisms are unclear. In this paper, we review the mechanisms underlying the role of EBV in the development of central nervous system diseases, which could bebeneficial in providing new research ideas and potential clinical therapeutic targets for neurological diseases.

## Introduction

Epstein-Barr virus (EBV) is an approximately 172-kb DNA, double-stranded gamma herpesvirus that contains a DNA-wrapped cyclic protein as the core, a nucleocapsid consisting of 162 capsules, a protein envelope located between the nucleocapsid and the envelope, and an outer envelope with an external virus-encoded membrane glycoprotein. There are two types of EBV, EBV1 and EBV2, which differ in the sequence of genes encoding EBV nuclear antigens (EBNA-2, EBNA-3A/3, EBNA-3B/4 and EBNA-3C/6). The virus infects B cells and nasopharyngeal epithelial cells in most immunologically active individuals. There are two states of latent infection and lytic replication in the host, which lead to differential gene expression. Some of the viral latent genes are not translatable, such as the RNA encoded by EBV (EBER1, EBER2), and some of the lytic genes such as BART and BHRF1 encode microRNAs that are involved in the process of EBV infection. Of course, most of the genes in the EBV genome can be translated into functional proteins such as BERF, BZLF, BKRF1, BNLF1, and BNRF0. However, the vast majority are in a long-term or even lifelong state of latent infection (Magaki et al., 2017; Zuo et al., 2019; Polepole et al., 2021). During latent infection, the commonly expressed proteins include six nuclear antigens (EBV nuclear antigen [EBNA] 1, EBNA2, EBNA3A, EBNA3B, EBNA3C, and EBNALP), three membrane-associated proteins (latent membrane protein [LMP] 1, LMP2A, LMP2B), and two small non-coding RNAs (EBER1 and EBER2) (Kerr, 2019; Li et al., 2020). EBNA1 (EBV nuclear antigen 1) plays a central role in maintaining viral genome replication and transcriptional regulation throughout the life cycle of the virus and is expressed in all EBV-infected cells (Marcucci and Obeidat, 2020; Varvatsi et al., 2021). It binds to the viral genome of latent origin and completes viral self-replication within the host cells, which is favorable for establishing infection (Sivachandran et al., 2012; Marcucci and Obeidat, 2020). In addition to their transforming activity and promotion of B-cell growth, LMP1 and LMP2A protect B-cells from apoptosis *via* B-cell receptor signaling (Rajcani et al., 2014). In B cells, virus lytic replication can occur during latency reactivation, and the transition from latency to the lytic cycle is mediated by BZLF1 and BRLF1 viral proteins that trigger the expression of certain viral genes (e.g., BCRF1, BHRF1) and down-regulate the host immune response, which ultimately leads to cell death and the release of viral particles (Cordier-Bussat et al., 1993; Baumforth et al., 1999). During lytic replication viral gene expression including glycoprotein 350/220, BZLF-1, BRLF1 promotes reactivation and triggers lytic replication through infected lymphocytes through the circulation to the central nervous system (CNS) (Jha et al., 2015; Houen and Trier, 2020; Lupia et al., 2020).

Studies have shown that EBV can replicate in the CNS and disrupt the integrity of the blood-brain barrier (BBB). BBB injury is associated with neurocognitive impairment, neuronal damage, and inflammation. EBV latently infected memory B-cells enter the lysis cycle *via* early viral proteins BZLF1 and BRLF1, leading to the release of viral particles that enter the CNS through infected lymphocytes and the blood circulation and induce pathogenesis (Meyding-Lamade and Strank, 2012; Van Gent et al., 2014; Liu and Cohen, 2016). A previous study demonstrated that EBV can cause neurological disease even in the absence of acute EBV infection, as evidenced by the detection of BZLF1 mRNA in most EBV patients with polymerase chain reaction (PCR)-positive cerebrospinal fluid (CSF) samples, indicating active EBV replication in the CNS(Lee et al., 2021). Thus, EBV-infected cells, free viruses, and gene products can be found in the CNS, and abnormally high EBV titers and positive EBV immunoglobulin G (IgG) or EBV IgM can be detected in CSF samples from patients with neurological damage (Shafiq et al., 2011; Lee et al., 2021). In addition, EBV can directly infect neurons, such as NT-2 neuronal cells, and RT-qPCR and immunofluorescence data show elevated expression of EBNA1 and EBNA3C and increased activity of lysis markers gp350 and BZLF1, suggesting that EBV is capable of lytic infection in neuronal cells (Jha et al., 2015). Other studies have also found that EBV infection may lead to advanced neurodegenerative disease, and intrathecal EBV reactivation may be a mechanism of inflammation underlying neurological disease. However, the mechanism of action is unclear (Kleines et al., 2011). In conclusion, EBV has a close relationship with the development of neurological diseases. This article reviews the role of EBV in the pathogenesis of neurological diseases and provides the potential molecular targets for the diagnosis, treatment, and prognosis of EBV-related neurological diseases.

## EBV and Alzheimer’s Disease

Alzheimer’s disease (AD) is a multifactorial, common, complex, and severe neurodegenerative disorder that primarily affects older adults and is characterized by progressive cognitive decline accompanied by a decline in motor function. The main pathological hallmarks are the aggregation of amyloid-beta peptides forming extracellular plaques and aggregation of hyperphosphorylated tau forming intracellular neurofibrillary tangles with neuroinflammation, gliosis, and neurodegeneration (Talwar et al., 2019; Allnutt et al., 2020; Andrews et al., 2020). Viral infections may be risk factors for susceptibility to AD (Kittur et al., 1992). Researchers have found that different viruses may involve different pathways and have varying distributions in different brain regions, and infection-related factors can enter the brains of older adults inducing chronic inflammation, leading to neurodegeneration (Carbone et al., 2014; Talwar et al., 2019).

Some microscopic pathological changes in AD are similar to those of chronic lentiviral disease, and viral infection may alter cell surface proteins and facilitate cell fusion. Thus, the viral hypothesis was proposed for the development of AD (Kittur et al., 1992). Histological findings from early studies suggested the presence of herpesvirus infection in neurofibrillary tangles and the hippocampus (Kittur et al., 1992). Serologic EBV positivity in patients with AD and EBV IgG plasma levels is associated with cognitive deterioration and clinical AD progression (Carbone et al., 2014; Allnutt et al., 2020). In AD patients with latent EBV infection, cognitive decline or memory impairment may stress dementia-susceptible individuals, which can lead to immune dysregulation and ultimately, EBV reactivation or replication. During a 5-years follow-up period, amnestic mild cognitive impairment (aMCI) events were significantly associated with elevated plasma anti-EBV IgG antibody concentrations and a greater proportion of EBV-positive peripheral blood leukocytes in older adults with clinical AD, suggesting a potential role for EBV in the pathogenesis of cognitive decline. Therefore, the EBV antibody concentration can be used as a biomarker to assess the risk of developing aMCI, suggesting the involvement of chronic EBV infection in the development of AD (Fagundes et al., 2014; Shim et al., 2017).

Genetic data from genome-wide association studies on AD suggest that the concurrence of single nucleotide polymorphisms (SNPs) can lead to genetic traits predisposing to AD *via* complex and diverse mechanisms, while each SNP increases an individual’s susceptibility to herpes virus infection (Carbone et al., 2014). Ilaria et al. found that most AD patients with EBV-positive brain tissue were APOEε4 carriers and had high levels of EBV-positive blood leukocytes. The APOEε4 allele in CTR increases EBV positivity, suggesting that the APOEε4 allele can influence EBV latency in older adults and increase brain susceptibility to the virus. This implies that the presence of EBV in the peripheral blood may be a risk factor for AD (Carbone et al., 2014; Kang and Liu, 2020).

**FIGURE 1:**
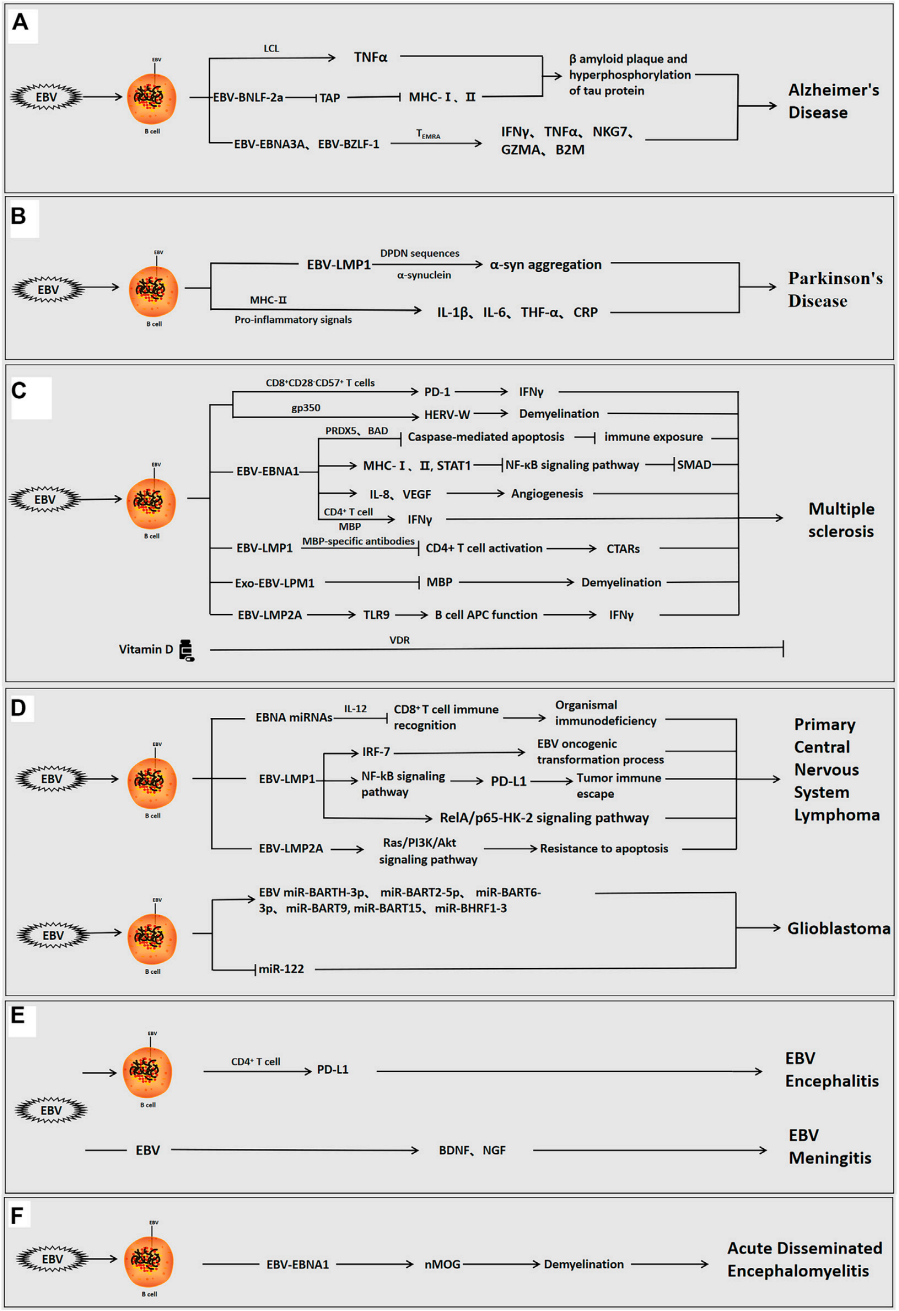
The role of Epstein-barr virus in neurological diseases. EBV can infect the organism directly, or indirectly *via* infection of B cells interact with host cells *via* its own encoded proteins (EBNA1, LMP1, LMP2A, etc.) and miRNAs to promote inflammation and regulate the immune response, and participate in the development of neurological disorders. **(A)** EBV-positive/infected B cells used the viral proteins to induce β-amyloid aggregation and tau protein hyperphosphorylation, cause neuroinflammation and modulate the immune response, and promote the development of AD. **(B)** EBV-infected B cells used the viral proteins to induce α-syn aggregation, cause neuroinflammation, and promote the development of PD. **(C)** EBV-positive B cells used the viral proteins to induce angiogenesis, neuroinflammation, immune escape and demyelination and facilitate the development of MS. In MS, vitamin D can also inhibit disease progression. **(D)** EBV**-**positive B cells used the viral proteins or viral miRNAs to induce immune escape, inhibit apoptosis, cause neuroinflammation, and promote the occurrence of brain tumorigenesis. **(E)** EBV can directly invade the organism or infect B cells to enter the brain, and viral proteins can then induce immune escape, cause neuroinflammation, and stimulate the development of encephalitis and meningitis. **(F)** EBV infection can induce demyelination, cause neuroinflammation, and promote the development of acute disseminated encephalomyelitis.

EBV causes neuroinflammation *via* infected peripheral blood mononuclear cells and brain monocytes/macrophages, which allow the virus to cross the BBB and replicate in the endothelial cells of the brain, leading to a loss of neurons in the white matter, and perfusion (Kittur et al., 1992; Kanakry et al., 2016). Tumor necrosis factor (TNF)-α is a cytokine, and decreased TNF-α can reduce the hyperphosphorylation of amyloid plaques and tau protein (Dezfulian, 2018). In AD, the lymphoblastoid cell line (LCL) in which EBV immortalizes of B-cells, high expression of TNF-α induces amyloid β-protein aggregation and tau protein hyperphosphorylation, thereby promoting the development of AD (Ounanian et al., 1992; Dezfulian, 2018). A recent study published by Gate et al. showed that the immune response was triggered by EBNA3A and BZLF-1 antigens. CD8^+^ T effector memory CD45RA^+^(T_EMRA_) cells were associated with immune memory and negatively correlated with cognitive performance. In AD, adaptive immune responses are mediated by CD8^+^ T_EMRA_ cells, and EBNA3A and BZLF1 interact with TEMR to release pro-inflammatory interferon-γ (IFNγ), TNF-α, and cytotoxic factors (NKG7, GZMA, and B2M), leading to decreased cognitive function and further exacerbation of AD (Carbone et al., 2014; Gate et al., 2020; Kang and Liu, 2020; Tiwari et al., 2021). In addition, EBV-encoded BNLF-2a blocks the transporter associated with antigen processing (TAP). BNLF-2a can further induce AD by inhibiting TAP and downregulating major human histocompatibility complex (MHC)-I and II expression, leading to the accumulation of neuronal cells and viral polypeptides in the environment (Figure 1A) (Tiwari et al., 2021).

During its latency and reactivation phases, EBV can stimulate a systemic stress immune response, which induces inflammation and, consequently, cognitive decline during aging (Carbone et al., 2014; Shim et al., 2017). However, there are few studies on the pathogenesis of EBV in AD, and further research is needed. This area of research should be a focus of future studies and may help to better elucidate the role of viral infection in the pathogenesis of AD.

## EBV and Parkinson’s Disease

Parkinson’s disease (PD) is another common neurodegenerative disorder of aging caused by a combination of genetic and underlying environmental factors. The main pathological features of PD are degeneration of nigrostriatal dopaminergic neurons, reduction of striatal dopamine, and the formation of abnormal protein aggregates in neurons, such as Lewy vesicles. The aging nervous system is susceptible to the direct and indirect effects of infection, and bacterial or viral infections are considered a potential risk factor (Pajares et al., 2020; Hirsch and Standaert, 2021; Smeyne et al., 2021). Predominantly lymphocytic leukocytosis, mildly increased protein levels, and EBV antibodies in the CSF and serum of patients with PD suggest the involvement of EBV infection in the development of PD (Bu et al., 2015; Schroder et al., 2018).

Epidemiological studies have demonstrated that patients with PD are significantly more seropositive for EBV than the general population. Latent EBV infection can trigger autoantibodies that can cross-react with α-synuclein and elevate α-synuclein aggregation (Woulfe et al., 2002; Bu et al., 2015). LMP1 is a virally encoded membrane protein expressed during EBV latency and is an important target for host cellular immune surveillance of latent EBV infection (Woulfe et al., 2000). EBV-induced anti-α-syn antibodies are at low levels throughout PD. LMP1 and α-syn share a similar protein primary sequence and consist of the amino acid PXDPDN. EBV-infected humans can produce anti-LMP1 antibodies against cross-reactive epitopes, and antibodies against the cross-reactive PQDPDN repeat region of the LMP1 protein have been identified in earlier assays. The DPDN sequence is a cross-reactive target of α-syn. In genetically susceptible individuals, the DDNGPQDPDN repeat region of LMP1 generates an immune response to the EDMPPVDPDN epitope of α-syn, which ultimately leads to α-syn aggregation *via* the cross-reactivity of autoantibodies. It is shown that LMP1 can interact with a-syn throughDPDNsequences to induce α-syn aggregation and further promote PD (Woulfe et al., 2014). More importantly, systemic inflammatory markers such as interleukin-1β(IL-1β), interleukin-6 (IL-6), TNF-α, and C-reactive protein (CRP) are associated with the risk of PD. During acute or persistent reactivation of infection, EBV can produce pro-inflammatory signals that promote antiviral responses *via* MHC-II risk alleles, leading to an autoimmune response and induction of PD (Figure 1B) (Sawada et al., 2014; Bu et al., 2015).

Despite the current progress in elucidating the pathogenesis of PD and the symptomatic treatment of PD-related symptoms, the mechanisms of EBV involvement in PD development are poorly understood, and further research is needed to develop relevant and effective treatments for improving and protecting neurons and delaying disease progression.

## EBV and Multiple Sclerosis

Multiple sclerosis (MS) is a common chronic inflammatory demyelinating disease of the CNS. Relapsing-remitting multiple sclerosis (RRMS) is the most common type of MS, and is the reason of non-traumatic neurological disability in young people. It is mainly characterized by multiple foci, remission, and relapse. The main pathological features are increased BBB permeability, leukocyte infiltration, demyelination, and neurodegeneration and reactive gliosis in the CNS, leading to progressive deterioration of sensory, motor, autonomic, and neurocognitive functions. It can be caused by various factors, such as environmental and genetic, as well as viral infections, and its etiology and pathogenesis are still unclear. (Laurence and Benito-Leon, 2017; Marquez et al., 2020; Ioannides et al., 2021).

EBV infection can induce organismal immune disorders and promote the development of MS (Hedstrom et al., 2020). The majority of adult patients with MS are EBV-positive before the onset of MS, indicating that EBV-seronegative individuals have a low risk of MS (Drosu et al., 2021). The elevated EBNA1-specific CD4^+^ T cells, which the CD4^+^ T cells contains a distinct specific epitope at the C-terminus of EBNA1. Compared to healthy seropositive individuals, patients with MS have elevated levels of EBNA1-specific CD4^+^ T cells, which secrete more inflammatory factor IFN-γ after cross-reacting with myelin antigens, which further induces the development of MS (Lunemann et al., 2006; Olsson, 2021; Zdimerova et al., 2021). In postmortem MS brain samples, CD8^+^ T-cells showed proliferative cytotoxic activity, induced T-cell infiltration, and released IFNγ, which can exacerbate demyelination (Abrahamyan et al., 2020; Veroni and Aloisi, 2021). Furthermore, the CD8^+^CD28^−^CD57^+^ T-cells have a central role in the immune response to EBV-induced MS. During EBV infection, the increased CD8^+^CD28^−^CD57^+^ T-cells upregulate PD-1 expression, increase IFN-γ release, inhibit the conversion of EBV-infected B-cells to memory cells and induce the development of MS (Cencioni et al., 2017). Moreover, EBV-infected B-cells and plasma cells accumulate in the brains of patients with MS (Soldan and Lieberman, 2020; Ioannides et al., 2021). Similarly, the accumulation of EBV-positive B-cells was observed patients with RRMS (Houen et al., 2020). Excessive proliferation of EBV-positive B-lymphocytes may be a risk factor for worsening MS, and the depletion of B-lymphocytes is a potential therapeutic strategy for MS (Figure 1C) (Fraser et al., 2015; Jog et al., 2020; Polepole et al., 2021).

In-depth studies have shown that EBV-encoded proteins (e.g., EBNA1, LMP1, LMP2A) derived from EBV-infected B cells are involved in the development of MS by inducing neuroinflammation and regulating the immune function of B and T cells (Tarlinton et al., 2019). Recent experiments revealed a significant correlation between EBV genes (EBNA-1, EBNA-2, EBNA-3B, LMP-1, and LMP-2A) and MS. Specifically, the EBNA3B1.2, EBNA3B2.1, and LMP1.1 alleles are positive related to the risk of MS. However, low expression of the LMP1.5, LMP2A4.2, EBNA1.3, EBNA2.1 and EBNA3B2.2 alleles had a protective effect against the development of MS (Varvatsi et al., 2021).

Compared with other viral proteins, there are many reports on the role of EBV-EBNA1 in MS. The concentrations of anti-EBNA1 antibodies in the CSF were significantly and negatively correlated with inflammatory factor interleukin-8 (IL-8) concentration relative to the total protein concentration, highlighting the role of IL-8 as an important mediator in the acute phase response in MS (Sisay et al., 2017). The most common abnormalities detected on CSF examination in patients with MS were increased intrathecal IgG synthesis and oligoclonal bands (OCBs) (Fernandez-Menendez et al., 2016). OCBs are oligoclonal IgG bands consisting of clonally restricted immunoglobulin detected by isoelectric focusing, and are important markers of persistent inflammation in several neuroinflammatory diseases and viral infections of the CNS (Yu et al., 2020). EBNA1 and EBNA2 concentrations are not only systemically elevated in patients with MS, but EBNA1 epitopes bind to CSF IgG and OCBs and are enriched in the CSF of patients with MS. Therefore, the interaction of EBNA1 epitopes and MS intrathecal antibodies corresponding to OCBs in the CSF suggests the potential involvement of EBV in the etiology of MS (Ruprecht, 2020; Wang et al., 2021).

The study by Hintzen et al. confirmed that serum EBNA1 IgG antibodies were higher in patients with MS than in healthy adults. Elevated EBNA1 IgG concentrations were associated with rs3135388 (HLA-DRB1*1501), rs2744148 (SOX8), rs11154801 (MYB), rs1843938 (CARD11), and rs7200786(CLEC16A/CIITA) SNP risk alleles in gene-gene interactions (Kreft et al., 2017). Notably, human leukocyte antigen (HLA)-DR is an MHC II protein. HLA-DRA encodes the HLA-DR β chain, and the most commonly HLA-DRB1, and the HLA-DRB1*1501 haplotype allele influences the risk of developing MS (Matern et al., 2020; Ramasamy et al., 2020). Further genome-wide association studies have identified more than 200 susceptibility genes, and the HLA allele HLA-DRB1*15:01 (HLA-DR15) acts synergistically with the immune response to EBV infection to increase the likelihood of developing MS by at least seven-fold. (Ruprecht, 2020; Menegatti et al., 2021; Zdimerova et al., 2021). Consortium et al. found that examination of the sera and CSF of MS patients revealed a positive correlation between EBNA1 concentrations and HLA DRB1*1501 (Zhou et al., 2016; Brutting et al., 2021). Antibodies against the EBNA1 GAR region, the anti-EBNA1 IgG at the C-terminus of this fragment (385–420), were found in the MS risk haplotype HLA-DRB1*1501 and deletion of the protective HLA-A*02 allele increased the risk of MS (Marcucci and Obeidat, 2020). However, the other mechanisms underlying this synergistic effect have not been identified.

Previous studied revealed that endogenous retroviruses (HERV) can induce autoimmune diseases associated with MS. EBV invades B-cells *via* the gp350 protein, which activates the expression of the HERV-W locus and triggers the demyelination process, leading to the development of MS(Hansen et al., 2017; Donati, 2020). Recent studies suggested that the high expression of EBNA-1 can upregulate MHC I and II and STAT1, inhibit the NF-κB pathway, downregulate SMAD, and induce the development of MS(Varvatsi et al., 2021). During latent and lytic infection, EBV inhibits caspase-mediated apoptosis *via* PRDX5 and BAD, leading to reduced EBNA1 immune exposure and the promotion of viral immune escape (Figure 1C) (Kreft et al., 2017).

It is well known that the immune response is closely related to inflammation (Cronkite and Strutt, 2018). MicroRNA (miR)-146a is mainly involved in the regulation of inflammation and is upregulated in the active brain lesions of MS. The combination of miR-146a and anti-EBNA-IgG significantly increases the risk of MS incidence and recurrence (Zhou et al., 2018). Angiotensin II(Ang-2) is a key component of angiogenesis and can regulate vasoendothelial growth factor (VEGF) production. The observations of an MS mouse model, as well as those of MS patients, revealed that Ang-2 concentrations were elevated in the neurons, glial cells, and inflammatory cells of the spinal cords of experimental autoimmune encephalomyelitis (EAE) mice. EBNA1 stimulated angiogenesis by promoting the secretion of inflammatory factor IL-8 and upregulating VEGF expression, thereby inducing the development of MS (Karampoor et al., 2016). In addition, soluble CD83 (sCD83) receptor concentrations were higher in MS patients than in healthy individuals. The serum sCD83 concentrations were negatively correlated with EBV antibody and vitamin D concentrations, suggesting that sCD83 may have antiviral properties and a role in preventing disease activity (Karampoor et al., 2018). Interestingly, vitamin D has anti-inflammatory effects and can reduce anti-EBNA1 IgG antibody concentrations *via* Vitamin D receptors (VDR), which can have a direct effect on B-cell function and slow the progression of MS (Figure 1C) (Marcucci and Obeidat, 2020; Teymoori-Rad et al., 2021).

It is worth noting that myelin basic proteins (MBPs) can bind not only to specific EBNA1 antibodies but also to the 411–426 epitope (Jog et al., 2020; Marcucci and Obeidat, 2020). Monoclonal MBP-specific antibodies in MS patients interact with EBV encoded LMP1 to suppress CD4^+^ T cell activation, leading to elevated antibody concentrations against CTARs and the development of MS (Lomakin et al., 2017). In addition, exosomes containing viral proteins (LMP1) from EBV-infected human oligodendrocyte lines were found to reduce MBP expression and promote demyelination (Sampey et al., 2014; Wu et al., 2020; Mrad et al., 2021). EBV-encoded membrane protein LMP2A in B cells is abnormally sensitive to TLR9 activation. TLR9 is associated with the progression of EAE, and TLR9 stimulation can enhance the APC function of B-cells. Therefore, LMP2A can also enhance B-cell APC function by activating TLR9, which activates the body’s immune response, promotes inflammation, releases inflammatory factor IFNγ, and further aggravates MS progression (Figure 1C) (Chang et al., 2012). However, studies investigating the role of LMP1/2A are lacking.

The above studies illustrate that EBV can interact with host cells *via* viral protein and non-coding RNA regulated by EBV to induce CNS inflammation, activate the immune response, and promote MS development, which involves key factors that can be used as potential biomarkers for diagnosis and treatment.

## EBV and Brain Tumors

EBV is an oncogenic virus that is closely associated with the development of various malignancies, including various brain tumors, such as primary CNS lymphoma (PCNSL) and glioma. EBV-encoded viral proteins (e.g., EBNA1, LMP1) and non-coding RNAs (miR-BARTs, miR-BHRFs, etc.) can promote tumor cell proliferation, differentiation, invasion, metastasis, immune escape, and anti-apoptosis (Price et al., 2018; E et al., 2020; Athanasiou et al., 2021; Gandhi et al., 2021; Tang and He, 2021).

PCNSLs account for approximately 2% of all primary CNS tumors, and the median age was approximately 65 years at diagnosis in a previous study (Hoang Oanh et al., 2020). PCNSL is an extra-nodal non-Hodgkin’s lymphoma confined to the brain, soft meninges, eyes, and spinal cord. The most common histologic type is diffuse large B-cell lymphoma, which is characterized as a highly aggressive high-grade B-cell tumor with a poor prognosis (Gandhi et al., 2021; Hajtovic et al., 2021). EBV DNA can be detected in the CSF of patients with PCNSL (Bathoorn et al., 2011). EBV-LMP1 genetic deletion and high EBNA2 expression are important pathogenic factors for EBV-positive PCNSLs in older immunocompetent patients (Sugita et al., 2018). The clinicopathological features of EBV-associated PCNSL are characterized by a cytotoxic T-cell phenotype, and it has been proposed that the pathogenesis of PCNSL involves a defect in the body’s specific immunity to EBV due to impaired cytotoxic T-cell activity. The peptide epitopes of the EBV EBNA2 antigen induce CD4^+^ T-cell responses following EBV infection in immunocompetent individuals, thereby inhibiting EBV-mediated B-lymphocyte proliferation (Ogura et al., 2013; Chatterjee et al., 2020). EBV microRNA-mediated downregulation of the cytokine IL-12 reduces the recognition of infected cells by EBV-specific CD8^+^ T-cells, which can further lead to an organismal immunodeficiency and induce morbidity (Sugita et al., 2018). In addition, EBV promotes malignant transformation by expressing anti-apoptotic genes, and the decreased immunocompetence can lead to the activation of previously latent EBV and subsequent proliferation of B-cells, thereby inducing PCNSL (Smith et al., 2007; Venetz et al., 2010; Lim et al., 2015; Hajtovic et al., 2021). Interferon regulatory factor 7 (IRF-7) is involved in the regulation of EBV latency and has oncogenic properties. In PCNSL, LMP1 immortalizes B-cells, induces IRF-7 expression, and activates IRF-7 *via* phosphorylation and nuclear translocation, thus enhancing the EBV oncogenic transformation process (Zhang et al., 2004). Further studies have indicated that LMP1 also activates the NF-kB signaling pathway, increases programmed death ligand 1 (PD-L1) promoter activity, and promotes tumor immune escape (Sethi et al., 2019). Knockdown of LMP1 leads to inactivation of the RelA/p65-HK-2 signaling pathway, thereby inhibiting tumor development (Tateishi et al., 2020). LMP2A, which is expressed during latent EBV infection, also induces B-cell immortalization and transformation and activates the Ras/PI3K/Akt signaling pathway, thus leading to apoptosis resistance, and induces the development of PCNSL (DeBoever et al., 2013) (Figure 1D). Unfortunately, the relationship between EBV and the poor prognosis of PCNSL has not been established.

Gliomas account for approximately 75% of malignant primary brain tumors, the vast majority of which are glioblastomas (GBMs). GBMs are fatal malignant tumors of the CNS with an approximate survival duration of 12–18 months after initial diagnosis (Lin et al., 2016; Daisy Precilla et al., 2021). EBV was detected in the brain tissue of patients with GBMs (Zavala-Vega et al., 2017). EBV RNA/DNA, miRNAs and proteins were also present in the plasma of GBM patients, and their EBV titers were significantly higher than those of controls. Furthermore, EBV-associated LMP1, LMP2, EBER, EBNA, and BART play important roles in the differentiation, proliferation, and apoptosis of tumor cells, suggesting that EBV is involved in the pathogenesis of GBM, although the mechanisms remain elusive (Zavala-Vega et al., 2019; Strojnik et al., 2017). Aberrantly expressed miRNAs can be detected in patients with gliomas (Karimzadeh et al., 2021). EBV miRNAs including miR-BARTH-3p, miR-BART2-5p, miR-BART6-3p, miR-BART9, miR-BART15, and miR-BHRF1-3 were found to be significantly elevated in the plasma of GBM patients (Limam et al., 2019). Host cellular miR-122 plays a tumor-suppressive role in the CNS, and EBV infection reduces miR-122 expression, leading to tumor progression (Karimzadeh et al., 2021). In GBM immunosuppressed individuals, EBV latent infection is activated to induce oncoprotein modification of the host-cells, thus promoting tumor progression (Figure 1D) (Fonseca et al., 2015).

Therefore, EBV is considered important in the development of gliomas. However, EBV is mainly closely related to the development of malignant tumors such as nasopharyngeal carcinomas and Burkitt’s lymphomas, and its mechanism in the pathogenesis of these tumors has been studied more frequently. There are fewer studies related to the molecular mechanism of EBV in PCNSLs and GBMs and additional investigation is needed.

## EBV in Encephalitis and Meningitis

Encephalitis includes viral encephalitis and viral meningitis. Viral encephalitis is a primary encephalitis caused by direct viral invasion of the brain parenchyma. The main clinical manifestations are brain parenchymal damage and intracranial hypertension. Viral meningitis is a diffuse inflammatory syndrome of the pia mater and arachnoid caused by various viral infections. Acute onset is more common, with clinical manifestations including fever, headache, vomiting, and signs of meningeal irritation (Dupuis et al., 2011; Venkatesan and Murphy, 2018; Aksamit, 2021). Aseptic meningitis is known to be closely associated with encephalitis, but its exact pathogenesis is unclear. The diagnosis is based on EBV heterologous IgM antibody tests, seropositivity of IgM for viral capsid antigens, and/or positive CSF PCR and imaging studies (Angelini et al., 2000; Rodrigo-Armenteros et al., 2019).

The pathogenesis of EBV encephalitis is related to direct EBV invasion of the CNS or an antibody-mediated post-infection inflammatory response (Rodrigo-Armenteros et al., 2019). The infiltration of the soft meninges or neural tissue by cytotoxic CD8^+^lymphocytes caused by viral infection or the deposition of antigen-antibody complexes may cause immune damage (Carman et al., 2013). Studies using a rat model of EBV encephalitis have shown that EBV can promote the development of encephalitis *via* transformed B-cells that secrete anti-neuronal antibodies or anti-EBV protein antibodies that cross-react with neuronal antigens (Tsuruyama et al., 2020). However, cytotoxic CD4^+^ activated memory T-cells in the CNS are associated with elevated PD-L1 expression. Further, EBV can suppress CNS immune responses by expressing viral proteins with immune evasion function (e.g, EBNA1, LMP1, LMP2A), thereby maintaining T-cell-mediated inflammatory responses and inducing the development of EBV encephalitis (Figure 1E) (Johnson et al., 2019; Lupia et al., 2020).

EBV meningitis can present with meningitis-like symptoms and diffuse edematous hemorrhage (Han et al., 2019). Serology and CSF resulting suggested that most patients present with reactivated EBV infection. Reactivation of EBV can occur *via* the establishment of persistent EBV infection in B-cells after primary infection. The reactivation of EBV in latent infected B cells occurs in two ways in the CNS: activation from abnormal lymphoid follicles, suggesting chronic CNS inflammation, and transplantation into the CNS from latently infected memory B-cells (Kleines et al., 2011; Kelly et al., 2012). Immunosuppressed patients have elevated concentrations of EBV antibodies in the blood due to large numbers of EBV-infected B-lymphocytes. If reactivation occurs in the circulation, the damaged blood-cerebrospinal fluid barrier in patients with meningitis can allow the virus to freely enter the CNS(Kelly et al., 2012). The transcription of the EBV lytic replication gene BZLF1 was also detected in the CSF of most immunocompetent patients, suggesting that EBV is actively replicating in immunocompetent patients (Weinberg et al., 2002; Bathoorn et al., 2011). Notably, the significantly elevated concentrations of BDNF and NGF were detected in patients with EBV-induced meningitis, leading to neuronal inflammation. This suggests that the upregulation of neurotrophic factors plays a key role in the inflammatory host response after EBV-induced ME, further indicating that EBV may play an important role in the mechanisms of neuronal inflammation and brain injury severity (Figure 1E) (Chiaretti et al., 2014). However, research on EBV in meningitis has mainly focused on clinical diagnosis, and the molecular mechanisms are less commonly studied. Further research is needed on this topic.

## EBV and Acute Disseminated Encephalomyelitis

Acute disseminated encephalomyelitis (ADEM) is an inflammatory demyelinating disease that can involve white matter and, in rare cases, gray matter lesions in acute or subacute states. It is common in children and is characterized by demyelinated lesions throughout the brain and spinal cord, and can even involve the basal ganglia, thalamus, and brainstem. The clinical features include acute onset, multifocal neurological deficits with a monophasic course, and a typical clinical course of rapid neurological deterioration over several days. Most patients have viral prodromal symptoms followed by the development of focal neurological deficits and a good prognosis after symptomatic treatment, although some cases experience severe complications and mortality (Bahadori et al., 2007; Phowthongkum et al., 2007; Di Carlo et al., 2011; Ali et al., 2019).

EBV is known to be associated with acute disseminated encephalomyelitis (Grillo et al., 2008). EBV can cause encephalopathy directly in the CNS or indirectly *via* autoimmune ADEM(Wong et al., 1999; Bahadori et al., 2007). The immune-mediated process of EBV-related acute disseminated encephalitis and infected encephalomyelitis can lead to the multifocal demyelination of peripheral venous white matter, positive EBV IgM in CSF, and positive EBV DNA in the serum (Saeed et al., 2014; Ali et al., 2019). One study found cross-reactivity between EBNA-1 and myelin oligodendrocyte glycoprotein (MOG), in which EBV infection triggered the production of anti-MOG antibodies thereby elevating MOG concentrations and inducing the development of ADEM (Selter et al., 2010; Lalive et al., 2011; Nakamura et al., 2017). Moreover, in immunosuppressed transplant patients with ADEM, local reactivation of EBV, triggers an inflammatory response, and aggravates the disease, demonstrating the association of EBV infection with the development of ADEM (Figure 1F) (Caucheteux et al., 2013).

## EBV and Acute Cerebellar Ataxia

Acute cerebellar ataxia is a paraneoplastic disease characterized by cerebellar swelling and mild cerebellar dysfunction. The clinical presentation is dominated by acute cerebellar ataxia with febrile illness, trunk ataxia, nystagmus, intentional tremor, headache, and altered mental status. The condition is more common in children and young adults. The prognosis is good, and most patients recover completely. Approximately 50% of clinical cases exhibit viral infection before the development of ataxia, but the exact pathogenesis is not clear (Muscat et al., 2017; Geurten et al., 2018; D'Ambrosio et al., 2020).

EBV infection can be the main manifestation of acute cerebellar ataxia (Ali and Lawthom, 2013). Patients are seropositive for EBV IgM and IgG, and OCBs of EBV viral capsid antigen antibodies can be found in the CSF(Al-Shokri et al., 2020; Soldan and Lieberman, 2020). EBV infection mainly affects the cerebral hemispheres and leads to hydrocephalus and increased intracranial pressure (Geurten et al., 2018). Studies demonstrate that the pathogenesis of cerebellar ataxia is related to direct EBV invasion of the CNS or a post-infection inflammatory response mediated by anti-neural antibodies (Ali and Lawthom, 2013; D'Ambrosio et al., 2020). In addition, EBV lytic replication in neurons during cerebellar ataxia is thought to contribute to lytic gene expression (e.g.BZLF1) and neuronal damage in the CNS(Soldan and Lieberman, 2020). Intravenous immunoglobulin relieves the symptoms and reduces the duration of cerebellar ataxia after EBV infection (Soldan and Lieberman, 2020). However, few studies have investigated on the pathogenesis of EBV in acute cerebellar ataxia and further studies are needed to demonstrate the role of other EBV-related proteins in this condition.

## Conclusion

Unfortunately, the pathogenesis of EBV in CNS disease is not fully understood, and there are few effective treatments. Some studies have reported that cimetidine can treat patients with chronic EBV reactivation, but its efficacy has not been confirmed in neurological disorders (Kerr, 2019). In addition, antiretroviral drugs (e.g., zidovudine and lamivudine) have been reported to induce long-term remission in MS, but the exact mechanism is not known (Maruszak et al., 2011; Chalkley and Berger, 2014; Skarlis et al., 2017). Further, valpromide, an amide derivative of valproic acid, inhibits the expression of BRLF1 and BZLF1 and, therefore, can inhibit some of the viral and cellular genes involved in EBV lysis infection, but there are few related studies and further validation is needed (Gorres et al., 2016). For cases of direct viral attack with or without a secondary immune response, antiviral therapy has the potential to delay disease progression and its combination with intravenous immunoglobulin therapy may enhance the treatment efficacy. Notably, exosomes are small extracellular vesicles with a diameter of 30–100 nm and include carrying proteins, nucleic acids, and lipids that mediate cell-to-cell signaling to regulate various functions of the host cell. Exosomes can serve as ideal carriers for the delivery of protein- or RNA-based therapeutic drugs to the brain and are potentially valuable biomarkers for the clinical diagnosis and treatment of diseases (Zhang et al., 2021). EBV can use exosomes containing viral proteins and various RNAs to cross the BBB, enter target cells through the exosome surface membrane proteins, bind to target cell surface proteins, transfer its cargo to the target cells, affect the signaling pathways of the target cells, trigger various responses in the target cells, and participate in the development of the CNS. However, studies on this topic are limited.

In general, EBV can enter the body *via* direct infection or indirectly *via* B-cell invasion, enter the CNS by regulating the function of B- or T-lymphocytes, and cause neuroinflammation and immune disorders, finally leading to the development of nervous system diseases. However, the molecular and pathogenic mechanisms underlying EBV infection need to be further investigated, and research in this area will be beneficial in providing better strategies for the diagnosis and treatment of EBV-related neurological diseases.

## Glossary

AD: Alzheimer’s disease
Ang-2: angiotensin II
ADEM: Acute Disseminated Encephalomyelitis
BBB: blood-brain barrier
CSF: cerebrospinal fluid
CRP: C-reactive protein
CNS: central nervous system
EBV: Epstein-barr virus
EAE: experimental autoimmune encephalomyelitis
EBNA: EBV nuclear antigen
GBMs: glioblastomas
HLA: human leukocyte antigen
IFN γ: interferon-γ
IL-1β: interleukin-1β
IL-6: interleukin-6
Ig: immunoglobulin
IRF: Interferon regulatory factor
LMP: latent membrane protein
MS: Multiple sclerosis
ME: meningitis
MHC: major histocompatibility complex
MOG: myelin oligodendrocyte glycoprotein.
MBP: myelin basic protein
OCBs: Oligoclonal bands
PD: Parkinson’s disease
PCNSL: primary central nervous system lymphoma
PCR: polymerase chain reaction
SNPs: single nucleotide polymorphisms
TNF: Tumor necrosis factor
T_EMRA_: T effector memory CD45RA^+^
VDR: Vitamin D receptor
VEGF: vasoendothelial growth factor
